# Coping with high advertising exposure: a source-monitoring perspective

**DOI:** 10.1186/s41235-022-00433-2

**Published:** 2022-09-05

**Authors:** Raoul Bell, Laura Mieth, Axel Buchner

**Affiliations:** Department of Experimental Psychology, Heinrich Heine University Düsseldorf, 40225 Düsseldorf, Germany

**Keywords:** Source memory, Source-monitoring framework, Advertising exposure, Consumer skepticism, Source credibility

## Abstract

Consumers are exposed to large amounts of advertising every day. One way to avoid being manipulated is to monitor the sources of persuasive messages. In the present study it was tested whether high exposure to advertising affects the memory and guessing processes underlying source attributions. Participants were exposed to high or low proportions of advertising messages that were intermixed with product statements from a trustworthy source. In a subsequent memory test, participants had to remember the sources of these statements. In Experiments 1 and 2, high advertising exposure led to increased source memory and decreased recognition of the statements in comparison to low advertising exposure. High advertising exposure also induced an increased tendency toward guessing that statements whose sources were not remembered came from advertising. The results of Experiment 3 suggest that the presence of advertising, relative to its absence, leads to a skeptical guessing bias. Being exposed to advertising thus has pronounced effects on the memory and guessing processes underlying source attributions. These changes in source monitoring can be interpreted as coping mechanisms that serve to protect against the persuasive influence of advertising messages.

## Introduction

Consumers are often annoyed by exposure to advertising that is perceived as excessive (Cho & Cheon, 2004). But how many advertising messages are consumers actually exposed to every day? It is surprisingly difficult to obtain scientifically reliable estimates for the average daily advertising exposure. According to the American Association for Advertising Agencies (Britt & Adams, 2007), many of the numbers that have been circulating through the media for decades are largely unsubstantiated. An empirically based estimate has been provided by Watkins et al. (2019) who equipped children with wearable cameras for 4 days that took photos every few seconds during waking hours. These photos were then analyzed to assess the total amount of advertising exposure. The results suggest that children in New Zealand between 11 and 13 years of age are exposed to an average of 638 advertisements every day which leads to an estimated number of about 233,000 advertising exposures per year. The authors note that this number probably underestimates the total advertising exposure because the images from the camera were not always clear enough to register all of the advertising messages. It can be expected that the exposure to advertising messages will continue to increase due to the rapid increase in the consumption of digital media (Twenge et al., 2019) and the increasing tendency of consumers to quickly switch between media contents (Yeykelis et al., 2014) and to use several media concurrently (Van der Schuur et al., 2015). Whatever the precise number of advertisements per day, it is clear that consumers are exposed to large amounts of advertising. This leads to the question of how high exposure to advertising affects information processing.

One challenge to information processing results from the perception that information from advertising sources often cannot be trusted. It is well established that the credibility of sources is primarily determined by two factors: expertise and trustworthiness (Pornpitakpan, 2004). It has long been known that trustworthiness decreases when there is a perceived self-interest to present information in a biased way. According to the Yale attitude change model (Hovland et al., 1953), “[o]ne of the most general hypotheses is that when a person is having a definite intention to persuade others, the likelihood is increased that he will be perceived as having something to gain and, hence, as less worthy of trust” (p. 23). Given that advertising can be defined as the business of persuading others, the trustworthiness of advertising must necessarily be low. Accordingly, surveys consistently provide data that “are largely consistent with the proposition that most consumers express an overall distrust of advertising” (Calfee & Ringold, 1994, p. 233). The assumption that advertising is not trustworthy is also echoed in modern theories on the consumers’ responses to advertising such as the persuasion knowledge model (Evans & Park, 2015; Friestad & Wright, 1994) which implies that “when individuals are exposed to a persuasive message they will activate and carry out strategies designed to defend against that persuasive message” (Evans & Park, 2015, p. 157). These strategies are referred to as coping strategies. Most of the research on the persuasion knowledge model is concerned with coping strategies that determine behavior at the time the persuasive message is processed. Here we examine the memory processes involved in coping with persuasion attempts.

Often there is a delay between the exposure to an advertising message and its effect on the consumer’s behavior. Over time, the message may become dissociated from the source. An alarming example of a source memory failure is that messages that were labeled as false at the time of encoding may later on be believed to be true because the messages are still recognized as familiar but their source is not recollected (Bacon, 1979; Begg et al., 1992; Skurnik et al., 2005). Furthermore, it has been observed that people tend to incorporate misinformation from fictional narratives into their real-world beliefs and attitudes even though they know that fiction is unreliable as a source of information, possibly because they fail to remember the source of the fictional information (Green & Brock, 2000). Source memory failures also serve as an important explanation of the sleeper effect (Hovland & Weiss, 1951; Hovland et al., 1953). The sleeper effect refers to the observation that the effect of a persuasive message may increase after a delay. A dominant explanation of this effect is that the persuasive intent of a communicator may function as a discounting cue at the time of encoding. Therefore, the impact of the message increases as the message becomes dissociated from its source over time. Given the central role of source memory in advertising effects, it seems important to gain a deeper understanding of how people monitor and remember the source of advertising messages.

There is, however, surprisingly little research in which source memory for advertisements is measured directly (but see Law, 1995, 1998, 2002). One exception is a recent study on source memory for advertisements that was concerned with advertisements mimicking the editorial content of the hosting media site (Bell et al., 2021). The hypotheses were based on the classical source-monitoring framework (Johnson, 1997; Johnson et al., 1993) according to which people often fail to explicitly remember the source of information and instead try to reconstruct the source based on whatever information is still available at retrieval. Naturally, the content of the message itself will contain cues to its source. Therefore, advertising statements that mimic trustworthy informational or scientific content will be attributed to an advertising source with a lower probability than typical advertising slogans (Bell et al., 2021).

However, source monitoring is not only influenced by the content of the message but also by the statistical structure of the learning environment (e.g., Bayen & Kuhlmann, 2011; Bell et al., 2020; Kuhlmann et al., 2012; Spaniol & Bayen, 2002). In the present study, we focus on the effects of advertising exposure on source monitoring by manipulating the proportion of advertising statements that were presented among trustworthy statements while holding the content of the messages constant between conditions. Given that there are so few prior studies that specifically deal with source memory for advertising messages, we have to rely on another area of research to generate hypotheses about source monitoring. Specifically, hypotheses can be derived from research on eyewitness testimonies because this is another area of research in which source credibility plays a crucial role. Studies in this area of research suggest that one coping mechanism for dealing with potential misinformation from untrustworthy sources is to increase source monitoring (e.g., Echterhoff et al., 2005; Lindsay & Johnson, 1989; Pena et al., 2017). For example, the exposure to a narrative from a source with a perceived self-interest to provide false information increases source monitoring just as much as an explicit instruction to learn the sources (Echterhoff et al., 2005). Pena et al. (2017) examined how exposure to different amounts of misinformation affects source monitoring. Participants watched a mock crime video and were exposed to a misleading narrative containing 20 %, 50 %, or 80 % misinformation. As expected, the misleading narrative caused a misinformation effect: Exposure to a high proportion of misinformation increased memory errors. However, a high proportion of misinformation was also associated with increased skepticism about the credibility of the source. The belief that the source was untrustworthy was associated with increased source monitoring, which reduced the negative effect of the false narrative on memory accuracy. These findings were interpreted as suggesting that a high proportion of misinformation leads participants to become more skeptical of the source, which, in turn, induces source monitoring as a coping strategy (Pena et al., 2017). This research thus allows to derive a first hypothesis: Given that advertising is an untrustworthy source, *high* e*xposure to advertising should lead to improved source memory*. However, given that eyewitness testimony is a completely different research domain, it is unclear whether the findings generalize to advertising. For example, it seems conceivable that achieving a high level of accuracy is perceived as particularly important when giving eyewitness testimony and less important when dealing with advertising. It is thus an empirical question as to whether or not source memory increases as a function of high advertising exposure.

However, it can be argued that relying on increased source monitoring is only of limited use as a coping strategy for dealing with high advertising exposure. It is well known from decades of research on source monitoring (Johnson et al., 1993; Mitchell & Johnson, 2009) that people often fail to veridically remember the context in which they have learned a particular piece of information so that source attributions are often based on guessing (Kuhlmann et al., 2021). With hundreds of advertising messages a day it is simply unrealistic to expect that consumers remember the source of every message. A full account of source attributions in advertising contexts must therefore explain how people arrive at source attributions when source memory fails. Source-monitoring measurement models (e.g., Batchelder & Riefer, 1990; Bayen et al., 1996; Bröder & Meiser, 2007; Meiser & Bröder, 2002) include guessing parameters that reflect mechanisms for arriving at source attributions when the source is not remembered. Guessing is not random because heuristic cues may bias guessing in one direction or the other. It is often assumed that the familiarity that is associated with a recognized statement provides a heuristic cue to the truth of the statement (Bacon, 1979; Begg et al., 1992). This recognition heuristic is useful if familiarity is a valid cue, that is, if consumers are exposed to more true than false statements, which is seen as one of the “tacit assumptions underlying the conduct of conversations in daily life” (Skurnik et al., 2005, p. 723). According to the framework of bounded rationality (Herzog & Hertwig, 2013), heuristics are useful because they reflect the statistical structure of the environment. If we are exposed to more true than false statements in everyday conversations, familiarity-based recognition is an ecologically rational cue to the truth of the statement. This ecological association between familiarity-based recognition and truth is called into question when we are exposed to a large number of advertisements because it can be argued that advertising, by exposing consumers to biased information, exploits the familiarity-based recognition heuristic with manipulative intent. High advertising exposure thus invalidates familiarity-based recognition as a cue for the truth of a statement. This raises the question of whether people attribute recognized statements to trustworthy sources even when they have been exposed to high levels of advertising, which would make them vulnerable to persuasive influences. However, there is evidence that people are able to flexibly apply recognition-based heuristics in appropriate contexts. For example, when people are instructed that all of the previously presented statements were false, recognition is used as a cue for falsehood and not as a cue for truth (Unkelbach & Stahl, 2009). When these principles are applied to source monitoring, it is possible to postulate that *high advertising exposure should induce a bias toward attributing recognized statements to advertising*. This hypothesis can be formally derived from the probability matching account (Bayen & Kuhlmann, 2011; Kuhlmann et al., 2012; Spaniol & Bayen, 2002) according to which source attributions reflect the statistical structure of the encoding environment. Source guessing relies on the learned probabilities of the sources. For example, if participants have experienced 80 % newspaper articles and 20 % advertisements, probability matching implies that participants should guess that statements whose source is not remembered were associated to advertising with a probability of 0.20.

However, source guessing may deviate to some degree from the results that can be expected based on probability matching. Systematic deviations may result, for example, from expectations and schematic biases that may deviate from the actual distribution of the sources in the learning environment (Bayen & Kuhlmann, 2011; Bayen et al., 2000; Bell et al., 2020, 2021; Kuhlmann et al., 2012; Marsh et al., 2006). According to the persuasion knowledge model (Evans & Park, 2015; Friestad & Wright, 1994), advertising may be met with skepticism. As argued above, advertising introduces uncertainty about the trustworthiness of information. This uncertainty may elicit *a skeptical bias toward guessing that statements whose source is not remembered came from advertising and not from a trustworthy source*. Such a skeptical guessing bias would be in line with the results of a recent study in which participants exhibited a stronger-than-expected bias toward guessing that statements were advertising statements when half of the statements presented during encoding were advertising statements (Bell et al., 2021). In the following, we define the skeptical guessing bias as a systematic deviation from those values that are expected when participants use probability matching. Specifically, we predict that participants should have a stronger bias toward guessing that statements whose sources are not remembered came from advertising than what can be expected based on probability matching.

## Experiment 1

The main purpose of Experiment 1 was to examine the effect of high versus low advertising exposure on memory for advertising and trustworthy sources as well as source guessing. Specifically, the experiment serves to examine (1) whether high advertising exposure leads to more source monitoring than low advertising exposure, (2) whether participants are more likely to guess that statements were advertising statements when advertising exposure was high rather than low, and (3) whether guessing conforms to what can be expected based on probability matching or whether guessing deviates from the expected values because participants show a skeptical guessing bias, that is, a higher-than-expected tendency to attribute statements whose source is not remembered to advertising.

### Method

#### Participants

The sample consisted of 93 participants (68 of whom were female) who were recruited on campus at Heinrich Heine University Düsseldorf before the COVID-19 pandemic. Their age ranged from 18 to 35 years with a mean age of 23 (SD = 4). Participants were seated individually in one of two sound-proof chambers. Upon arrival, participants were alternately assigned to the low-advertising-exposure condition (*n* = 47) or to the high-advertising-exposure condition (*n* = 46). With a total sample size of *N* = 93, *α* = 0.05, and 160 statements in the memory test, it was possible to detect effects of the size *w* = 0.03 in the multinomial analysis of the guessing parameters with a statistical power of 1 − *β* = 0.95 (Faul et al., 2007). Using population parameter values based on the parameter estimates of Experiment 1 by Bell et al. (2021) and assuming that the population parameter values of the guessing parameters under H1 are located symmetrically above and below 0.50 (assumed under H0), this corresponds to a difference in the guessing parameters between the low-advertising-exposure condition and the high-advertising-exposure condition of somewhat less than ∆*a* = 0.10 for the recognized items or somewhat less than ∆*g* = 0.20 for the unrecognized items (for an explanation of these parameters, see below).

#### Materials

For each individual participant, 160 product statements were randomly selected from a pool of 260 product statements that had been created for a previous study (Bell et al., 2021). The 260 statements had received neutral credibility ratings in the associated norming study (*M* = 0.00 on a seven-point scale ranging from − 3 [not credible at all] to + 3 [highly credible], SD = 0.62). Examples are “Ixt brand socks do not lose their good fit even after frequent washing”, “Bastol’s grape juice is made only from the best quality grapes”, and “The Calwell printer has low ink consumption”. The product statements contained 260 different brand names that were created using the pseudoword generator *wuggy* (Keuleers & Brysbaert, 2010). Examples are *Balen, Birle, Gemoo, Moxy, Pinx, Ulmi, Zomic*, and *Zürz*. The brand names were randomly assigned to the product statements.

#### Procedure

The procedure closely followed that of a recent study on schematic influences on source memory for advertisements (Bell et al., 2021). The encoding of the product statements and their sources was incidental. Participants were instructed that they would see statements about products. There were two sources from which these product statements could have emanated. The advertising statements were labeled as “Advertising”. The trustworthy source, the “Brand Testing Foundation”, was described as a respectable impartial research institute. In the exposure phase, 80 product statements were shown. The statements were written in black 32 pt *Times* font at the center of the screen of the computer that controlled the experiment.

**Fig. 1:**
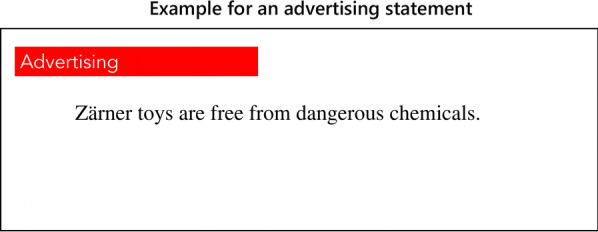
Example for an advertising statement. *Note*: The statement has been translated to English; the version used in the experiment was in German

In the low-advertising-exposure condition, 60 (75 %) of the statements were assigned to the trustworthy source and labeled “Brand Testing Foundation” and 20 (25 %) of the statements were assigned to the advertising source and labeled “Advertising.” In the high-advertising-exposure condition, 60 (75 %) of the statements were assigned to the advertising source and 20 (25 %) of the statements were assigned to the trustworthy source. The labels were shown in 29 pt *Avenir* font in the upper left corner of the frame in which the product statement was written (Fig. 1). The label “Advertising” was written in white font against a red background. The label “Brand Testing Foundation” was written in white font against a blue background. The difference in background color served to increase the perceptual distinctiveness of the labels.

For each participant, half of the 160 product statements were randomly selected for the exposure phase. These product statements were randomly assigned to the sources. The statements were presented in a different random order for each participant. The label appeared one second before the product statement was shown. The participants were asked to rate the credibility of each statement on a seven-point scale ranging from − 3 (not credible at all) to + 3 (highly credible). After a click on the “continue” button, the statement disappeared from the screen and, after an inter-trial interval of 1 s, the next trial started. A progress bar at the bottom of the screen showed the percentage of trials that had been completed.

After the exposure phase, 20 trials of a serial-recall task had to be completed as a distractor task. In each trial, a sequence of eight digits had to be remembered. The digits were randomly drawn from the set {1, 2 … 9} without replacement. Each digit was presented for 1 s in the middle of the computer screen. Immediately after the presentation of the last digit, eight question marks appeared on the screen. The participants’ task was to replace the question marks with the to-be-remembered digits by typing them in the correct order using the number pad of the computer keyboard. After each trial, participants received a feedback about the number of digits they had correctly remembered. The distractor task lasted about 10 min.

After the distractor task, participants received instructions for the surprise source memory test. In this test, each participant saw a different random sequence of the 80 statements from the exposure phase intermixed with 80 new statements. All statements were presented in black 30 pt *Arial* font against a white background in the center of the screen. Participants first rated the credibility of the statements on a seven-point scale ranging from − 3 (not credible at all) to + 3 (highly credible). Then they were required to indicate whether they had seen the statement before or not by clicking on “old” or “new “. If the statement was judged to be “old”, participants were asked to give a source judgment by attributing the statement to “Advertising” or to the “Brand Testing Foundation”. Participants confirmed each selection by clicking a “continue” button. This closely corresponds to the standard procedure of source memory tests (Bayen et al., 1996; Kuhlmann et al., 2021). A progress bar at the bottom of the screen showed participants the percentage of trials that was already completed.

### Results

**Fig. 2:**
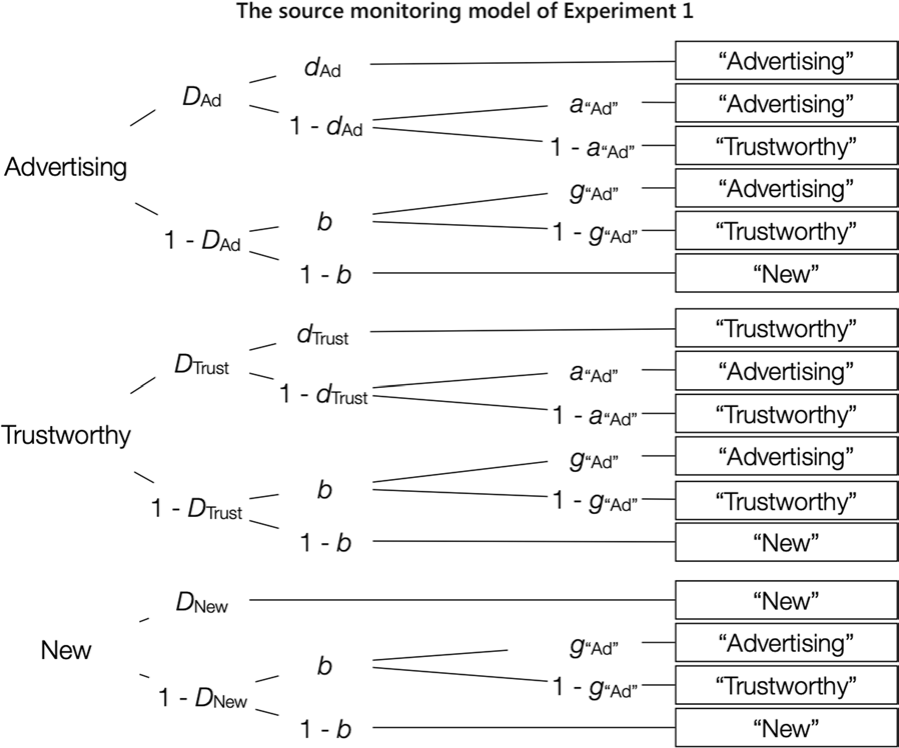
The source-monitoring model of Experiment 1. *Note*: The source-monitoring model of Bayen et al. (1996) was adapted to the present Experiment 1. Each model tree represents the cognitive processes in response to the advertising, trustworthy, and new statements that were presented in the source-monitoring test. The rectangles on the right represent the participants’ responses. Letters along the branches represent the latent cognitive processes which lead to these responses with certain probabilities (*D*_∙_ = statement recognition, *d*_∙_ = source memory, *b* = guessing that the statement had been presented in the exposure phase, *a*_“Ad”_ = guessing that a recognized statement was from the advertising source, *g*_“Ad”_ = guessing that an unrecognized statement was from the advertising source)

In the analyses reported here, we will focus on the model-based analyses of statement recognition and source memory for the advertising and trustworthy sources, as well as on the source-guessing parameters that are central to our hypotheses. Supplementary exploratory analyses of the data that are secondary to our research goals are provided at the OSF project page (see data availability section). Figure 2 shows the two-source model of source monitoring of Bayen et al. (1996), adapted to the present study. The upper tree of the model represents the processes that occur in response to the product statements from the advertising source. The parameters represent probabilities between 0 and 1. With probability *D*_Ad_, participants recognize the advertising statement from the exposure phase. If a statement is recognized, participants may remember the advertising source with probability *d*_Ad_, in which case the statement is correctly remembered as advertising. With the complementary probability 1 − *d*_Ad_, participants do not remember the source of the statement, in which case they may correctly guess, with probability *a*_“Ad”_, that the statement was from the advertising source, or, with the complementary probability 1 − *a*_“Ad”_, they may incorrectly guess that the statement was from the trustworthy source. If participants do not recognize the statement with probability 1 − *D*_Ad_, they may still guess, with probability *b*, that the statement had been presented in the exposure phase, in which case they may guess correctly, with probability *g*_“Ad”_, that the statement is from the advertising source, or they may guess incorrectly, with the complementary probability 1 − *g*_“Ad”_, that the statement is from the trustworthy source. With probability 1 − *b*, the statement is guessed to be new. Parallel processes occur in response to statements from the trustworthy source (middle tree of Fig. 2) and new statements (bottom tree of Fig. 2). To analyze the results, we need two instances of the model illustrated in Fig. 2, one for the low-advertising-exposure condition and one for the high-advertising-exposure condition.

The source-monitoring model is well-validated (Bröder & Meiser, 2007). It has been empirically demonstrated that the model parameters independently reflect item recognition, source memory and guessing processes (Bayen et al., 1996). For these reasons the model is widely used in source-monitoring research to distinguish between item recognition, source memory, and guessing (e.g., Bayen & Kuhlmann, 2011; Kuhlmann et al., 2012; Schaper et al., 2019a, b) and has recently been applied to examine schematic influences on source memory for advertisements (Bell et al., 2021).

The model displayed in Fig. 2 includes eight parameters that represent the to-be-measured cognitive processes (*D*_Ad_, *D*_Trust_, *D*_New_, *d*_Ad_, *d*_Trust_, *b*, *a*_“Ad”_, *g*_“Ad”_), but there are only six independent data categories to fit. Therefore, equality restrictions have to be imposed on the model to arrive at an identifiable base model (Bayen et al., 1996; Erdfelder et al., 2009). The base model used here includes the assumption that the detection of old statements does not differ from the detection of new statements and is not affected by source (*D*_Ad_ = *D*_Trust_ = *D*_New_). To ensure model identifiability, it is technically necessary to assume that the probability of detecting an old item as old is equal to the probability of detecting a new item as new. Validation studies have shown that models that incorporate this restriction perform comparable to signal-detection-based models and better than one-high-threshold models that deny that new items can be detected (Bayen et al., 1996; Komar et al., in press; Schütz & Bröder, 2011; Snodgrass & Corwin, 1988). As another requirement of model identifiability, we also included the assumption that source memory does not differ between the advertising and the trustworthy source (*d*_Ad_ = *d*_Trust_, referred to as *d* further on). This assumption is in line with evidence showing that there is no reliable memory advantage of untrustworthy over trustworthy sources (Bell et al., 2010, 2021; Henkel & Mattson, 2011; Nadarevic & Erdfelder, 2013, 2019; Schaper et al., 2019a, b). We thus used Model 5a of Bayen et al.’s taxonomy of identifiable submodels of the two-high threshold source-monitoring model. The base model with these restrictions fit the data of Experiment 1 well, *G*^2^(2) = 0.99, *p* = 0.61. This indicates that the assumptions incorporated in the base model were compatible with the data (for an additional empirical test of these assumptions, see Experiment 2). The model-based analyses were performed with multiTree (Moshagen, 2010).

#### Source memory

**Fig. 3:**
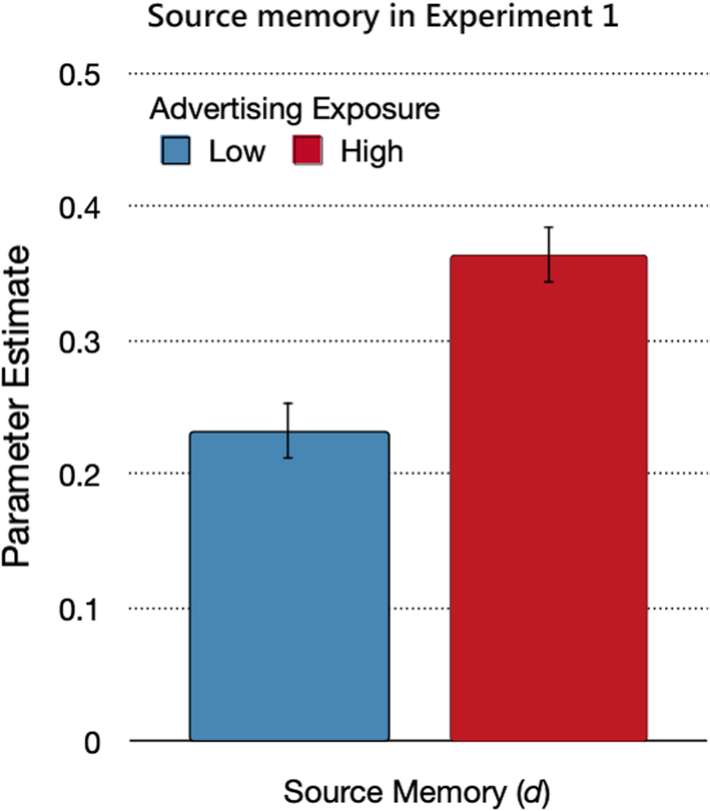
Source memory in Experiment 1. *Note*: Parameter estimates of the source memory parameter *d*, reflecting the probability of correctly remembering the source of the statements from the advertising and trustworthy sources, as a function of advertising exposure (low, high). The error bars reflect standard errors

The prediction that source monitoring increases as a function of advertising exposure translates into the hypothesis that parameter *d* should be higher under high advertising exposure than under low advertising exposure. This hypothesis was confirmed by the model-based analysis. Source memory was better when advertising exposure was high than when it was low (Fig. 3), Δ*G*^2^(1) = 19.79, *p* < 0.01, *w* = 0.04.

#### Source guessing

**Fig. 4:**
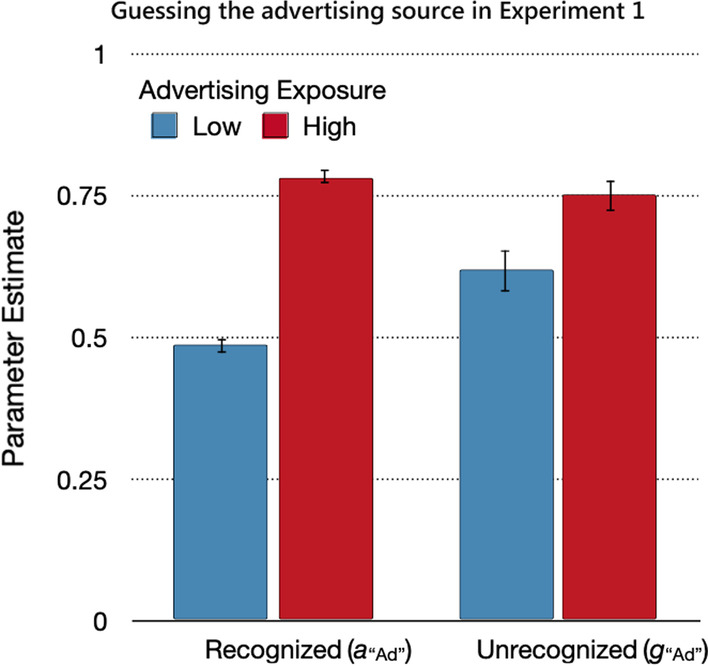
Guessing the advertising source in Experiment 1. *Note*: Parameter estimates of the parameters reflecting the probabilities of guessing the advertising source for recognized (*a*_“Ad”_) and unrecognized (*g*_“Ad”_) statements as a function of advertising exposure (low, high). The error bars represent standard errors

The probability of guessing that statements were from the advertising source (Fig. 4) differed between recognized and unrecognized statements, Δ*G*^2^(2) = 11.18, *p* < 0.01, *w* = 0.03. High advertising exposure was associated with an increased tendency toward guessing that statements whose sources were not remembered were from the advertising source. The effect of advertising exposure on source guessing was more pronounced for recognized statements, Δ*G*^2^(1) = 201.64, *p* < 0.01, *w* = 0.12, than for unrecognized statements, Δ*G*^2^(1) = 8.61, *p* < 0.01, *w* = 0.02. The guessing parameters were compared against those values that were expected based on probability matching. Participants in the high-advertising-exposure condition showed a bias toward guessing the advertising source that exceeded the expected value of 0.75 for recognized statements, Δ*G*^2^(1) = 6.94, *p* < 0.01, *w* = 0.02, and matched the expected value of 0.75 for unrecognized statements, Δ*G*^2^(1) < 0.01, *p* > 0.99, *w* < 0.01. By contrast, participants in the low-advertising-exposure condition did not show a corresponding bias toward guessing that the statement came from the trustworthy source. The probability of guessing that statements were from the advertising source stayed above the expected value of 0.25 for both recognized, Δ*G*^2^(1) = 380.20, *p* < 0.01, *w* = 0.16, and unrecognized, Δ*G*^2^(1) = 115.09, *p* < 0.01, *w* = 0.09, statements.

#### Statement recognition

Statement recognition was worse when advertising exposure was high than when it was low, Δ*G*^2^(1) = 10.24, *p* < 0.01, *w* = 0.03. The full set of estimates of the parameters that could be derived from the source-monitoring model is reported in Table 1.

**Table 1:** Parameter estimates for item memory for the statements, source memory, and guessing as a function of advertising exposure (low, high) in Experiment 1

	Low advertising exposure		High advertising exposure	
*D*_Ad_ = *D*_Trust_ = *D*_New_	.81	(0.01)	.78	(0.01)
*d*_Ad_ = *d*_Trust_	.23	(0.02)	.36	(0.02)
*b*	.27	(0.02)	.30	(0.02)
*a*_“Ad”_	.49	(0.01)	.78	(0.01)
*g*_“Ad”_	.62	(0.04)	.75	(0.03)

Parameter *D*_∙_ represents the probability of recognizing statements as old or new. Parameter *d*_∙_ represents the conditional probability of remembering the sources of recognized statements. Guessing parameter *b* represents the probability of guessing that unrecognized statements are old. Parameters *a*_“Ad”_ and *g*_“Ad”_ represent the conditional probabilities of guessing that recognized or unrecognized statements were labeled as advertising. Values in parentheses represent standard errors

### Discussion

As expected, source memory was improved under high advertising exposure compared to low advertising exposure. Furthermore, high advertising exposure induced a tendency toward guessing that a statement came from the advertising source. This tendency was more pronounced for statements that were recognized than for statements that were not recognized.

## Experiment 2

The main purpose of Experiment 2 was to test whether the findings of Experiment 1 can be replicated conceptually, that is, under somewhat different conditions. Against the backdrop of the discussion about the robustness of psychological findings (Open Science Collaboration, 2015), we consider such a test of replicability an important prerequisite for drawing clear inferences. Furthermore, a limitation of Experiment 1 was that the assumption that source memory does not differ between advertising and trustworthy sources was necessary for obtaining an identifiable model and thus cannot be tested against the data. Experiment 2 does not have this limitation because this assumption is not needed for obtaining identifiability of the model variant used in Experiment 2. The main difference to Experiment 1 is that we additionally included, as a third category, statements from an unlabeled source. Nevertheless, the same ratio of trustworthy to untrustworthy information was used in the low-advertising-exposure (3:1) and the high-advertising-exposure (1:3) conditions.

### Method

#### Participants

The sample consisted of 73 participants (46 of whom were female) who were recruited on campus at Heinrich Heine University Düsseldorf. Their age ranged from 18 to 37 years with a mean age of 25 (SD = 5). Written informed consent was obtained from all participants. Up to five participants were seated in individual cubicles constructed with sound-absorbing walls. The participants wore over-ear headphones with high-insulation hearing protection covers to shield them from distraction. Upon arrival, participants were alternately assigned to the low-advertising-exposure condition (*n* = 36) or the high-advertising-exposure condition (*n* = 37). With a sample size of *N* = 73, *α* = 0.05, and 150 statements in the memory test, it was possible to detect small effects of the size *w* = 0.03 with a statistical power of 1 − *β* = 0.95 in the multinomial analysis of the guessing parameters.

#### Materials and procedure

Materials and procedure were identical to those of Experiment 1 with the following exceptions. In the exposure phase, 75 product statements were shown. In the high-advertising-exposure condition, 45 of these statements were labeled as advertising, 15 were associated with the trustworthy source, and 15 were unlabeled. In the low-advertising-exposure condition, participants saw 45 statements of the trustworthy source, 15 advertising statements and 15 statements from the unlabeled source. In the source memory test, participants saw the 75 statements from the exposure phase, randomly intermixed with 75 new statements. If a statement was judged as old, the participants were asked to give a source judgment by answering whether the statement originated from the advertising source, the trustworthy source, or the unlabeled source.

### Results

**Fig. 5:**
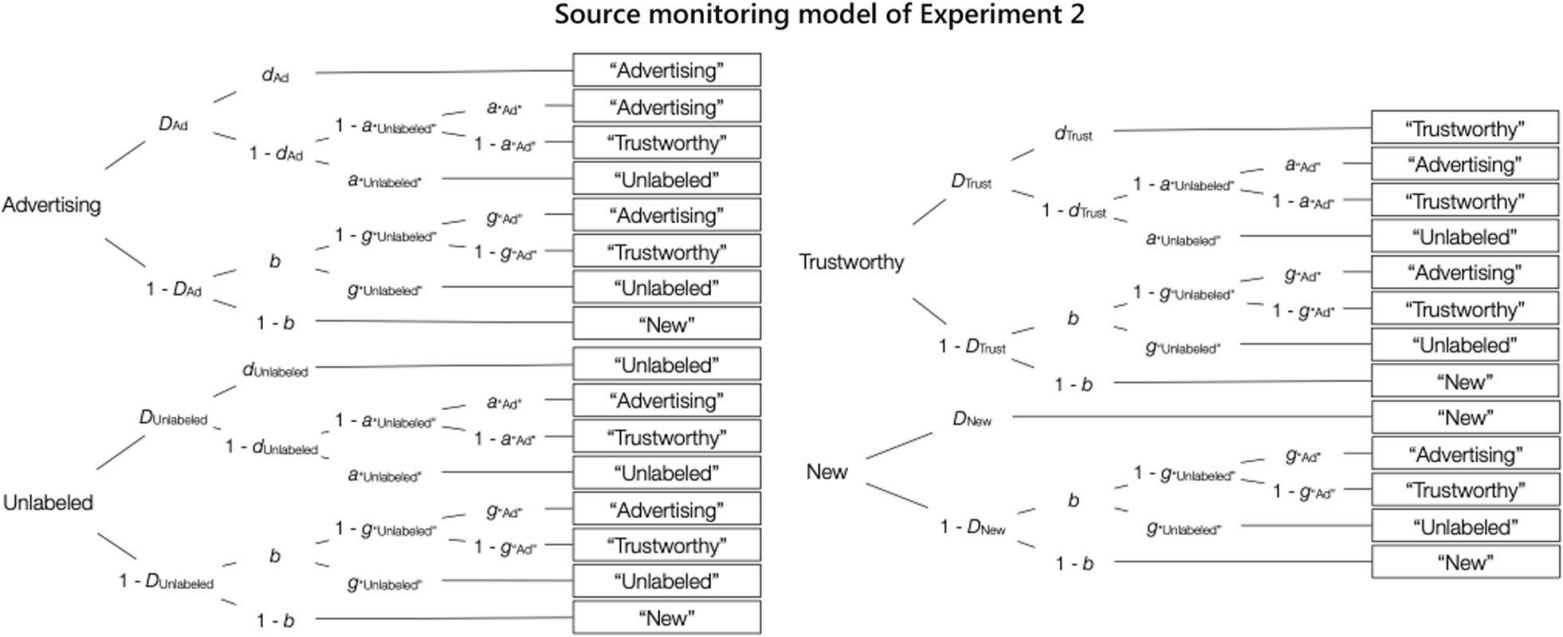
Source-monitoring model of Experiment 2. *Note*: The model was adapted to Experiment 2 from the three-source source-monitoring model of Keefe et al. (2002). Each tree represents the cognitive processes in response to the advertising, unlabeled, trustworthy, and new statements that were presented in the source-monitoring test. The rectangles on the right represent the participants’ responses. Letters along the branches represent the latent cognitive processes which lead to these responses with certain probabilities (*D*_∙_ = statement recognition, *d*_∙_ = source memory, *b* = guessing that the statement was from the exposure phase, *a*_“Unlabeled”_ = guessing that a recognized statement was presented without source label, *a*_“Ad”_ = guessing that a recognized statement was from the advertising source, *g*_“Unlabeled”_ = guessing that an unrecognized statement was presented without source label, *g*_“Ad”_ = guessing that an unrecognized statement was from the advertising source)

Due to the inclusion of a third source (the unlabeled source), Experiment 2 required an analysis with the three-source variant of the source-monitoring model (Keefe et al., 2002) which is well established in source-monitoring research (e.g., Buchner et al., 2009; Kroneisen et al., 2015; Nadarevic & Erdfelder, 2013, 2019). Figure 5 shows the model, adapted to the present study. In comparison to the two-source model used in Experiment 1 (Fig. 2), the three-source model displayed in Fig. 5 comprises four more parameters (*D*_Unlabeled_ for recognizing unlabeled statements, *d*_Unlabeled_ for remembering that the statement was presented without label, *a*_“Unlabeled”_ for guessing that a recognized statement was presented without label, *g*_“Unlabeled”_ for guessing that an unrecognized statement was presented without label). There are also six additional independent data categories from which the parameters can be estimated. It is thus possible to start with a base model with fewer assumptions compared to the base model of Experiment 1. In fact, only one assumption is required to obtain an identifiable three-source model: The probability of detecting unlabeled statements is set to be equal to the probability of detecting new statements (*D*_Unlabeled_ = *D*_New_), which corresponds to the standard assumption of two-high threshold models (Bayen et al., 1996; Snodgrass & Corwin, 1988). This base model fit the data well, *G*^2^(2) = 0.24, *p* = 0.89. The assumption that recognition does not differ between advertising and the trustworthy source was compatible with the data, as was the assumption that source memory does not differ between advertising and the trustworthy source, Δ*G*^2^(4) = 5.26, *p* = 0.26, *w* = 0.02. These assumptions could thus be incorporated into a new base model incorporating the same assumptions as the base model in Experiment 1. This new base model fit the data well, *G*^2^(6) = 5.50, *p* = 0.48, and was used to obtain the parameter estimates and to perform the hypothesis tests.

#### Source memory

**Fig. 6:**
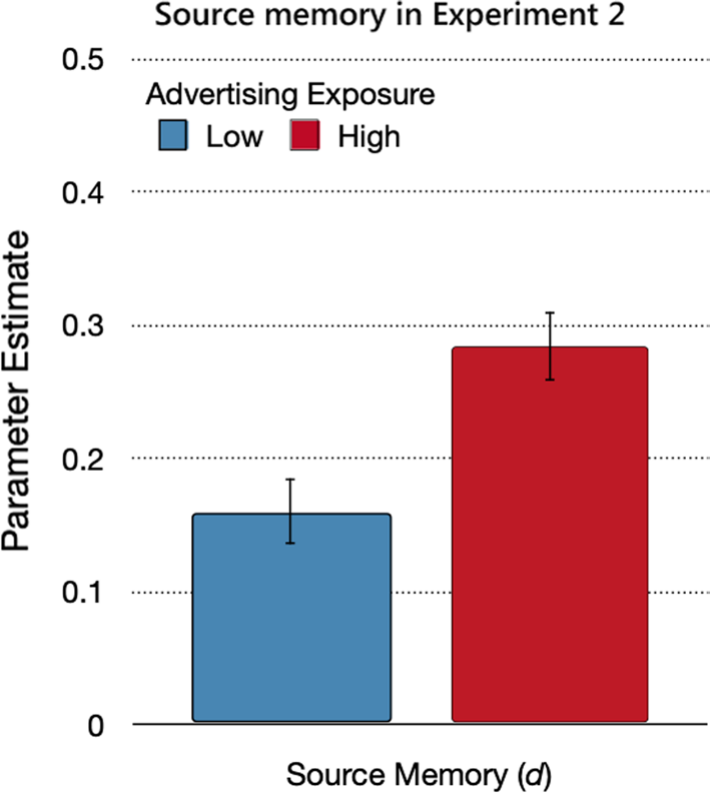
Source memory in Experiment 2. *Note*: Parameter estimates of the source memory parameter *d*, reflecting the probability of correctly remembering the source of the statements from the advertising and trustworthy sources, as a function of advertising exposure (low, high). The error bars reflect standard errors

Source memory for the advertising and trustworthy sources was better when advertising exposure was high than when it was low (Fig. 6), Δ*G*^2^(1) = 11.96, *p* < 0.01, *w* = 0.03.

#### Source guessing

**Fig. 7:**
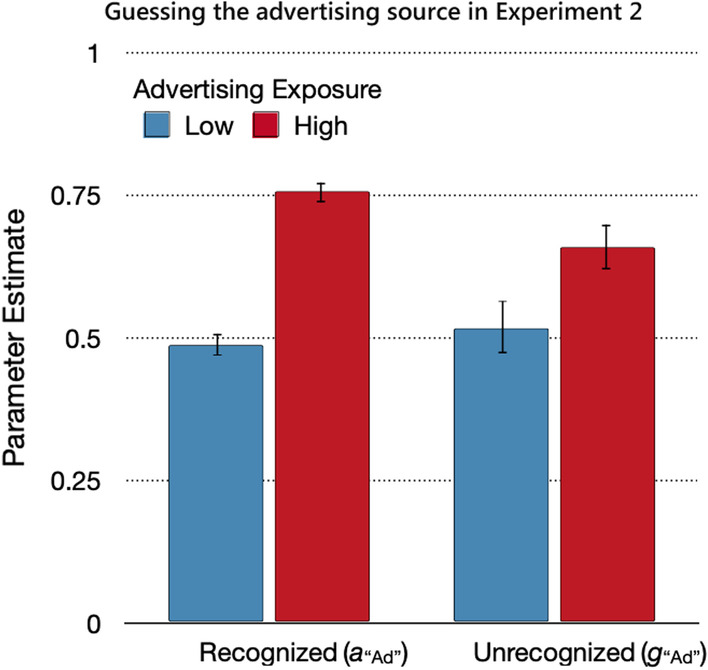
Guessing the advertising source in Experiment 2. *Note*: Parameter estimates of the parameters reflecting the probabilities of guessing the advertising source for recognized (*a*_“Ad”_) and unrecognized (*g*_“Ad”_) statements as a function of advertising exposure (low, high). The error bars represent standard errors

The probability of guessing that statements were from the advertising source (Fig. 7) differed between recognized and unrecognized statements, Δ*G*^2^(4) = 18.20, *p* < 0.01, *w* = 0.04. The high-advertising-exposure condition was associated with a higher probability of guessing that a statement was from the advertising source than the low-advertising-exposure condition. The effect of advertising exposure on source guessing was more pronounced for the recognized statements, Δ*G*^2^(1) = 103.59, *p* < 0.01, *w* = 0.10, than for the unrecognized statements, Δ*G*^2^(1) = 5.24, *p* = 0.02, *w* = 0.02.

The guessing parameters were compared against those values that were expected based on probability matching. Participants in the high-advertising-exposure condition showed a bias toward guessing the advertising source that corresponded to the expected value of 0.75 for recognized statements, Δ*G*^2^(1) = 0.16, *p* = 0.69, *w* < 0.01, and underestimated the expected value of 0.75 for unrecognized statements, Δ*G*^2^(1) = 6.09, *p* = 0.01, *w* = 0.02. By contrast, participants in the low-advertising-exposure condition showed no corresponding bias toward guessing that the statement came from the trustworthy source. The probability of guessing that a statement’s source was advertising exceeded the expected value of 0.25 for both recognized, Δ*G*^2^(1) = 201.40, *p* < 0.01, *w* = 0.14, and unrecognized, Δ*G*^2^(1) = 37.96, *p* < 0.01, *w* = 0.06, statements.

#### Statement recognition

Recognition of the statements from the advertising and trustworthy sources was again worse when advertising exposure was high than when it was low, Δ*G*^2^(1) = 17.75, *p* < 0.01, *w* = 0.04. The full set of estimates of the parameters that could be derived from the source-monitoring model is reported in Table 2.

**Table 2:** Parameter estimates for statement recognition, source memory, and guessing as a function of advertising exposure (low, high) in Experiment 2

	Low advertising exposure		High advertising exposure	
*D*_Ad_ = *D*_Trust_	.78	(0.01)	.69	(0.02)
*D*_Unlabeled_ = *D*_New_	.74	(0.02)	.70	(0.02)
*d*_Ad_ = *d*_Trust_	.16	(0.02)	.28	(0.03)
*d*_Unlabeled_	.08	(0.04)	.04	(0.04)
*b*	.24	(0.02)	.30	(0.02)
*a*_“Unlabeled”_	.27	(0.01)	.28	(0.02)
*g*_“Unlabeled”_	.31	(0.04)	.42	(0.03)
*a*_“Ad”_	.49	(0.02)	.76	(0.02)
*g*_“Ad”_	.52	(0.05)	.66	(0.04)

Parameter *D*_∙_ represents the probability of recognizing statements as old or new. Parameter *d*_∙_ represents the conditional probability of remembering the sources of recognized statements. Parameter *b* represents the probability of guessing that unrecognized statements were old. Parameters *a*_“Unlabeled”_ and *g*_“Unlabeled”_ represent the conditional probabilities of guessing that recognized or unrecognized statements were presented without label. Parameters *a*_“Ad”_ and *g*_“Ad”_ represent the conditional probabilities of guessing that recognized or unrecognized statements were labeled as advertising. Values in parentheses represent standard errors

### Discussion

Experiment 2 confirms the conclusions that were drawn from Experiment 1. As expected, source memory for the advertising and trustworthy sources was better when advertising exposure was high than when it was low. Furthermore, high advertising exposure induced a pronounced tendency toward guessing that a statement had an advertising source. This tendency was more pronounced for statements that were recognized than for statements that were not recognized.

However, the probability of guessing that a statement originated from advertising rather than from the trustworthy source did not directly correspond to the ratio of advertising versus trustworthy statements in Experiments 1 and 2. Participants in the high-advertising-exposure condition showed a strong tendency toward guessing the advertising source. By contrast, participants in the low-advertising-exposure condition did not show a corresponding tendency toward guessing the trustworthy source. Therefore, the question arises as to why participants were so reluctant to guess the trustworthy source in the low-advertising-exposure condition. A possible explanation is that this deviation from probability matching (Bayen & Kuhlmann, 2011; Kuhlmann et al., 2012; Spaniol & Bayen, 2002) is due to a skeptical guessing bias. If there is a possibility that a statement whose source is not remembered might have originated from advertising, participants may display a higher-than-expected tendency to guess advertising rather than the trustworthy source unless this is already their dominant guessing strategy. This assumption is in line with the results of a previous study (Bell et al., 2021) in which participants showed a higher-than-expected tendency toward guessing the advertising source even when the statements had a credible content. However, this previous study, just as the present Experiments 1 and 2, lacks a control condition in which advertising is absent. To test the hypothesis that the presence of advertising elicits a skeptical guessing bias, Experiment 3 contrasts a condition in which advertising was present with a control condition in which advertising was absent.

## Experiment 3

In Experiment 3, we presented statements from two research institutes. In an advertising-absent condition the research institutes were both described as trustworthy while in the advertising-present condition one of the research institutes was described as trustworthy while the other was described as an advertising agency that publishes pseudo-scientific rankings. The main purpose of Experiment 3 was to test whether participants in the latter condition would show a skeptical guessing bias reflected in a tendency to attribute statements whose sources were not remembered to advertising. As in Experiment 2, we also included a third source without labels to be able to test the critical assumption that source memory does not differ between advertising and trustworthy sources against the data.

### Method

#### Participants

The sample consisted of 179 participants (133 of whom were female) who were recruited on campus at Heinrich Heine University Düsseldorf. Their age ranged from 18 to 38 years with a mean age of 22 (SD = 3). Written informed consent was obtained from all participants. Up to five participants were seated in individual cubicles that were constructed with sound-absorbing walls and wore over-ear headphones with high-insulation hearing protection covers to shield them from distraction. Upon arrival, participants were alternately assigned to the advertising-absent condition (*n* = 90) or the advertising-present condition (*n* = 89). A larger sample size than in Experiments 1 and 2 was chosen because we anticipated the effects of advertising absence versus presence on source guessing to be smaller than the effects of probability matching observed in Experiments 1 and 2. With a sample size of *N* = 179, *α* = 0.05, and 150 statements in the memory test, it was possible to detect small effects of the size *w* = 0.02 with a statistical power of 1 − *β* = 0.95 in the multinomial analysis of the guessing parameters.

#### Materials and procedure

A norming study was conducted to select two names for research institutes with an a-priori neutral credibility. To this end, 43 names of fictional research institutes such as “Consumer Research Laboratory” or “Institute for Quality Testing” were created. In the norming study, participants were instructed that they would see names of research institutes conducting product tests and comparisons. They were told that some of the names referred to trustworthy independent research institutes that met the highest scientific standards and acted in the interests of consumers while other names referred to untrustworthy market research institutes that published pseudo-scientific rankings on behalf of the advertising industry and deliberately misled consumers in the process. Participants were then asked to rate, without much deliberation, whether the names of the institutes made a non-credible or credible first impression using a seven-point scale ranging from − 3 (not credible at all) to + 3 (highly credible). Based on the ratings of 17 participants, two names with the same a-priori neutral credibility—“Association for Product Testing” (*M* = 0.24, SD = 1.60) and “Institute for Innovation and Transparency” (*M* = 0.24, SD = 1.44)—were selected.

Materials and procedure of Experiment 3 were identical to those of Experiment 2 with the following exceptions. Participants had to rate the credibility of product statements that came from one of two known sources or from a third unlabeled source. The instructions differed between the advertising-present condition and the advertising-absent condition. In the advertising-present condition, the instructions read:

The first source is [Institute 1], a respectable research institute. [Institute 1] is an impartial foundation that works independently and is funded with state money. [Institute 1] is fully committed to the consumers’ well-being and advocates for high quality standards, product safety, sustainability, and environmental protection. Independent reviews repeatedly come to the conclusion that [Institute 1] meets the highest scientific standards and is dedicated to the interests of the customers.

The second source is [Institute 2], a dubious market research company. [Institute 2] belongs to a fraudulent advertising agency that works on behalf of the advertising industry. [Institute 2] provides paid advertising for products and does not care about quality standards, product safety, sustainability, or environmental protection. Independent reviews show that [Institute 2] publishes pseudo-scientific rankings and has knowingly misled customers.

In the advertising-absent condition, both sources were described as respected research institutes whereas in the advertising-present condition only the first institute was trustworthy while the second institute was described as a source of advertising. The first institute was thus always trustworthy while the second institute was the advertising source in the advertising-present condition and served as a trustworthy-control source in the advertising-absent condition. In both conditions, the names of the institutes were randomly assigned to either the first or the second source. Participants were further informed that the source was sometimes unlabeled. They were then asked to rate, on a seven-point scale ranging from − 3 (not credible at all) to + 3 (highly credible) how credible they thought the statements were depending on the content and source. In the exposure phase, 75 statements were shown. Of these 75 statements, 25 statements were assigned to Institute 1, 25 statements were assigned to Institute 2, and 25 statements were presented without a label. The label “Association for Product Testing” was written in white 29 pt *Avenir* font in front of a red background while the label “Institute for Innovation and Transparency” was written in white 29 pt *Avenir* font in front of a blue background.

In the source memory test, participants saw the 75 statements from the exposure phase, randomly intermixed with 75 new statements. When a statement had been judged as old, participants were asked to give a source judgment by answering whether a statement came from the “Association for Product Testing”, the “Institute for Innovation and Transparency”, or from the unlabeled source.

### Results

To analyze the results of Experiment 3, we used the three-source model (Fig. 5), adapted to the present study. Parallel to the analysis of Experiment 2, we started with a model that only implies that the detection of statements with unlabeled source is equal to the detection of new statements (*D*_Unlabeled_ = *D*_New_). This model fit the data well, *G*^2^(2) = 0.58, *p* = 0.74. The assumption that the recognition of statements did not differ between the trustworthy source and the advertising or trustworthy-control sources was compatible with the data, as was the assumption that source memory does not differ between the trustworthy source and the advertising or trustworthy-control sources, Δ*G*^2^(4) = 2.85, *p* = 0.58, *w* = 0.01. These equality assumptions could thus be incorporated into a new base model, which thus is based on the same assumptions as the base model used in Experiment 2. This new base model fit the data well, *G*^2^(6) = 3.43, *p* = 0.75. Parallel to Experiment 2, this base model was used to obtain the parameter estimates and to perform the hypotheses tests.

#### Source memory

**Fig. 8:**
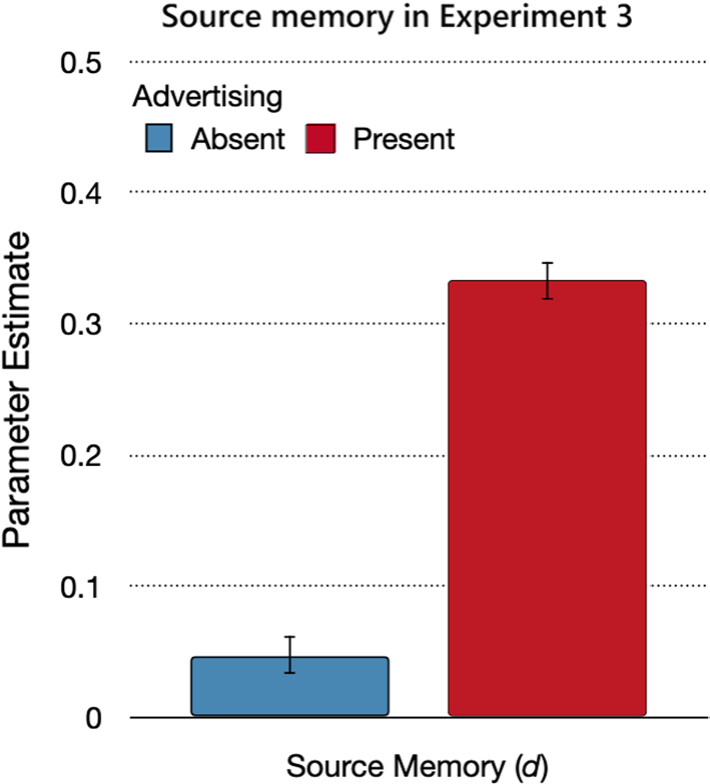
Source memory in Experiment 3. *Note*: Parameter estimates of the source memory parameter *d*, reflecting the probability of correctly remembering the source of the statements from the trustworthy and advertising sources. The error bars reflect standard errors

Source memory for statements from the trustworthy source and the advertising or trustworthy-control sources was significantly higher in the advertising-present condition than in the advertising-absent condition (Fig. 8), Δ*G*^2^(1) = 194.53, *p* < 0.01, *w* = 0.09.

#### Source guessing

**Fig. 9:**
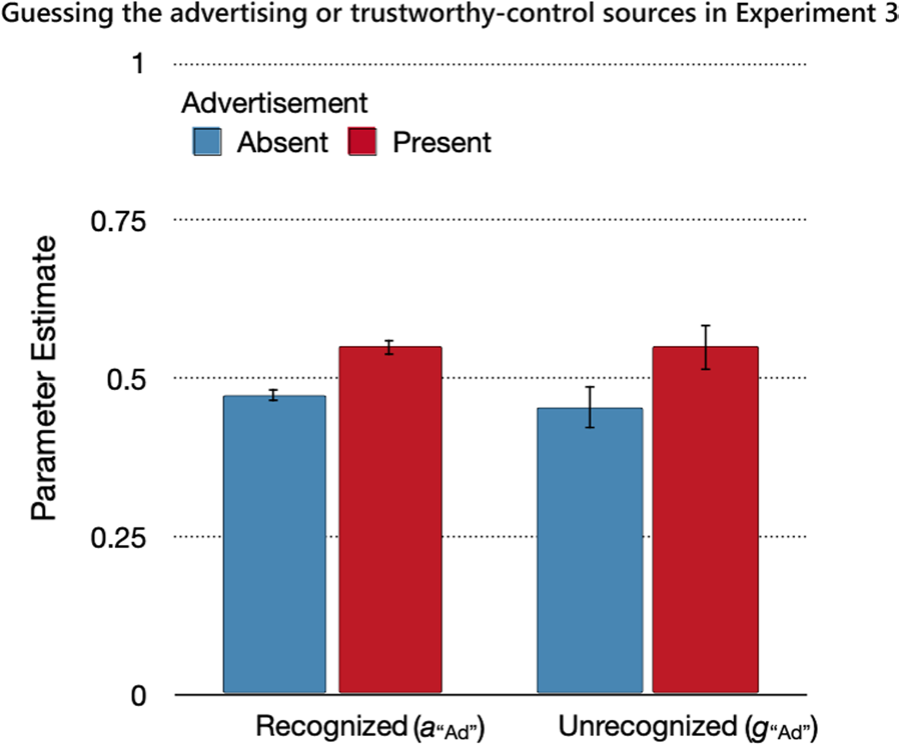
Guessing the advertising or trustworthy-control sources in Experiment 3. *Note*: Parameter estimates of the parameters reflecting the probabilities of guessing that recognized and unrecognized statements were from the advertising source (advertising present) or trustworthy-control source (advertising absent). The error bars represent standard errors

Guessing that a statement for which the source was not remembered belonged to the advertising or trustworthy-control sources did not differ between recognized and unrecognized statements, Δ*G*^2^(2) = 0.24, *p* = 0.89, *w* < 0.01. This equality assumption could be incorporated into a new base model for the analysis of the guessing parameters, which fit the data well, *G*^2^(8) = 3.67, *p* = 0.89. Participants in the advertising-present condition guessed with a higher probability that a statement was from the advertising source than participants in the advertising-absent condition guessed that a statement was from the trustworthy-control source, Δ*G*^2^(1) = 31.94, *p* < 0.01, *w* = 0.03 (Fig. 9). Given that the two labeled sources were presented with the same probability, the expected value based on probability matching is 0.50. The guessing bias underestimated this value when advertising was absent, Δ*G*^2^(1) = 9.63, *p* < 0.01, *w* = 0.02, but exceeded this value when advertising was present, Δ*G*^2^(1) = 22.59, *p* < 0.01, *w* = 0.03.

#### Statement recognition

Recognition of the statements from the trustworthy source and the advertising or trustworthy-control sources did not differ as a function of whether advertising was present or absent, Δ*G*^2^(1) = 2.31, *p* = 0.13, *w* = 0.01. The full set of estimates of the parameters that could be derived from the source-monitoring model is reported in Table 3.

**Table 3:** Parameter estimates for statement recognition, source memory, and guessing as a function of advertising presence (absent, present) in Experiment 3

	Advertising absent		Advertising present	
*D*_Ad/Control_ = *D*_Trust_	.77	(0.01)	.76	(0.01)
*D*_Unlabeled_ = *D*_New_	.75	(0.01)	.79	(0.01)
*d*_Ad/Control_ = *d*_Trust_	.05	(0.01)	.33	(0.01)
*d*_Unlabeled_	.26	(0.02)	.03	(0.03)
*b*	.23	(0.01)	.23	(0.01)
*a*_“Unlabeled”_	.30	(0.01)	.43	(0.01)
*g*_“Unlabeled”_	.47	(0.03)	.45	(0.03)
*a*_“Ad”/“Control”_	.48	(0.01)	.55	(0.01)
*g*_“Ad”/“Control”_	.46	(0.03)	.55	(0.04)

Parameter *D*_∙_ represents the probability of recognizing statements as old or new. Parameter *d*_∙_ represents the conditional probability of remembering the sources of the statements. Guessing parameter *b* represents the probability of guessing that unrecognized statements were old. Parameters *a*_“Unlabeled”_ and *g*_“Unlabeled”_ represent the conditional probabilities of guessing that recognized or unrecognized statements were presented without label. Parameters *a*_“Ad”/“Control”_ and *g*_“Ad”/“Control”_ represent the conditional probabilities of guessing that recognized or unrecognized statements belonged to the advertising or trustworthy-control sources. Values in parentheses represent standard errors

### Discussion

The main purpose of Experiment 3 was to test whether participants would show a tendency toward guessing that statements whose sources were not remembered came from the advertising source. The results confirm this hypothesis. If one of the sources was trustworthy and the other was untrustworthy, participants showed a tendency to guess that statements whose sources were no longer remembered came from the advertising source. This provides further evidence of a skeptical guessing bias.

An interesting question is how the present results were influenced by the fact that participants gave credibility judgments in Experiments 1 to 3. There are two possibilities worth considering. The first possibility, brought up by a reviewer during the review process, is that participants actually do not remember whether a source was trustworthy or not but instead remember only whether or not they gave high or low ratings. Fortunately, this hypothesis can be easily put to an empirical test: If the hypothesis is true, then source memory should be eliminated, or should at least be strongly reduced, in an experiment without credibility ratings. Examining the role of credibility ratings is also interesting for a second reason: The present study is concerned with coping mechanisms when confronted with advertising but people may show resistance to persuasion attempts in some situations more than in others. Internet users are often exposed to covert persuasion attempts that may be less likely to activate persuasion knowledge and coping mechanisms (Evans & Park, 2015). For example, when browsing social media for entertainment and inspiration, people may often fail to spontaneously scrutinize the credibility of information and may, in consequence, be likely to fall for manipulation attempts (Pennycook et al., 2020, 2021). There is a surge of research on how to motivate Internet users to activate their cognitive immune system against online misinformation and persuasion attempts. One intervention that has been proven effective in reducing the reliance on misinformation is to simply encourage readers to critically evaluate the accuracy of news statements or fictional texts (Epstein et al., 2021; Pennycook et al., 2020, 2021; Rapp et al., 2014). It is thus possible to interpret the requirement to evaluate the credibility of the product statements in Experiments 1 to 3 as a manipulation that may have activated or amplified some of the coping mechanisms of which evidence was observed in terms of increased source monitoring. It is thus interesting to examine the degree to which participants spontaneously engage in these coping mechanisms when they simply read product statements and what their sources are (similar to purposelessly browsing a website) rather than being required to scrutinize the credibility of these statements. To the extent that participants *spontaneously* engage in source monitoring when being confronted with an abundance of advertising, we expect Experiment 4 to replicate the findings of the previous experiments. However, if some or all of these mechanisms are only applied when the task requires participants to evaluate the credibility of information, the evidence in favor of these mechanisms of the cognitive immune system should be either absent or considerably weaker in Experiment 4.

## Experiment 4

The main purpose of Experiment 4 was to test whether the effects of advertising exposure on memory for the advertising and trustworthy sources and source guessing that were observed in Experiments 1 and 2 can be replicated when participants are not asked to provide credibility ratings of the statements at encoding and test.

### Method

#### Participants

The sample consisted of 89 participants (77 of whom were female). Their age ranged from 18 to 55 years with a mean age of 24 (SD = 7). Due to the COVID-19 pandemic, Experiment 4 was performed online and made available to the participants via www.soscisurvey.de (Leiner, 2019). The participants were recruited by advertising the experiment via email, in social media, and on the homepage of the Department of Experimental Psychology in Düsseldorf. All but one of the participants had at least a university entrance qualification, which suggests that the level of education was similar to that of the students who participated in Experiments 1 to 3. Participants were only allowed to participate with computers or laptops, not with smartphones or tablets. Participants were asked to complete the experiment alone in a quiet environment. They were asked to close all other browser windows and computer programs and to turn their smartphones off. Informed consent was obtained prior to participation. Participants were informed that they could withdraw their consent at any time and were asked at the end of the experiment to confirm that their data could be included in the data analysis. Of those participants who started reading the product statements, ten did not complete their participation (6 in the low-advertising-exposure condition, 4 in the high-advertising-exposure condition). Participants were alternately assigned to the low-advertising-exposure condition (*n* = 44) or the high-advertising-exposure condition (*n* = 45). With a sample size of *N* = 89, *α* = 0.05, and 160 statements in the memory test, it was possible to detect small effects of the size *w* = 0.03 with a statistical power of 1 − *β* = 0.95 in the multinomial analysis of the guessing parameters.

#### Materials and procedure

Materials and procedure were identical to those of Experiment 1 with the following exceptions. To use only web-safe fonts, the statements were written in 24 pt *Times New Roman* font and the advertising labels were written in 24 pt *Arial* font. The labels were shown simultaneously with the statements. Participants were not required to give credibility judgments. Instead, they were simply informed that they were “going to read a series of product statements”. Each statement was presented for 5 s before a “continue” button appeared. Upon clicking this button, the next statement was shown. A progress bar at the top of the screen showed the percentage of trials that had been completed. As a distractor task, participants were asked to solve ten simple mathematical problems (e.g., 13 − 7 = ?). The distractor task lasted about 40 s. In the memory test, the statements were presented in 14 pt *Arial* font. Participants were not asked to provide credibility ratings in the test phase. Instead, they were directly asked, for each statement, whether the statement was “old” or “new”. If the statement was judged to be “old”, participants were asked to give a source judgment by attributing the statement to “Advertising” or to the “Brand Testing Foundation”. Participants confirmed each selection by clicking a “continue” button. A progress bar at the top of the screen showed the percentage of completed trials.

### Results

The results were analyzed using the two-source model of source monitoring by Bayen et al. (1996), adapted to the present study (Fig. 2). Other than in the previous experiments, the assumption that old-new recognition did not differ between advertising and the trustworthy source was not compatible with the data (see analysis below). We therefore incorporated only the assumptions *D*_Trust_ = *D*_New_ and *d*_Ad_ = *d*_Trust_ (Model 6a in the nomenclature of Bayen et al., 1996) to arrive at an identifiable base model. The base model is saturated with zero degrees of freedom which implies that only assumptions on top of the base model can be tested.

#### Source memory

**Fig. 10:**
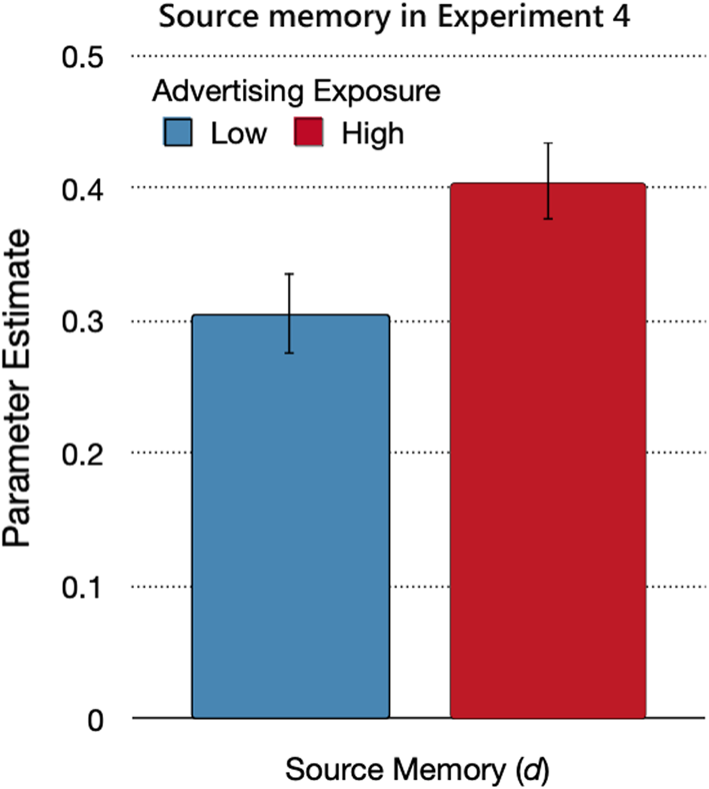
Source memory in Experiment 4. *Note*: Parameter estimates of the source memory parameter *d*, reflecting the probability of correctly remembering the source of the statements from the advertising and trustworthy sources, as a function of advertising exposure (low, high). The error bars reflect standard errors

The prediction that source monitoring increases as a function of advertising exposure translates into the hypothesis that parameter *d* should be higher under high advertising exposure than under low advertising exposure. This hypothesis was confirmed by the model-based analysis. Source memory was better when advertising exposure was high than when it was low (Fig. 10), Δ*G*^2^(1) = 5.72, *p* = 0.02, *w* = 0.02.

#### Source guessing

**Fig. 11:**
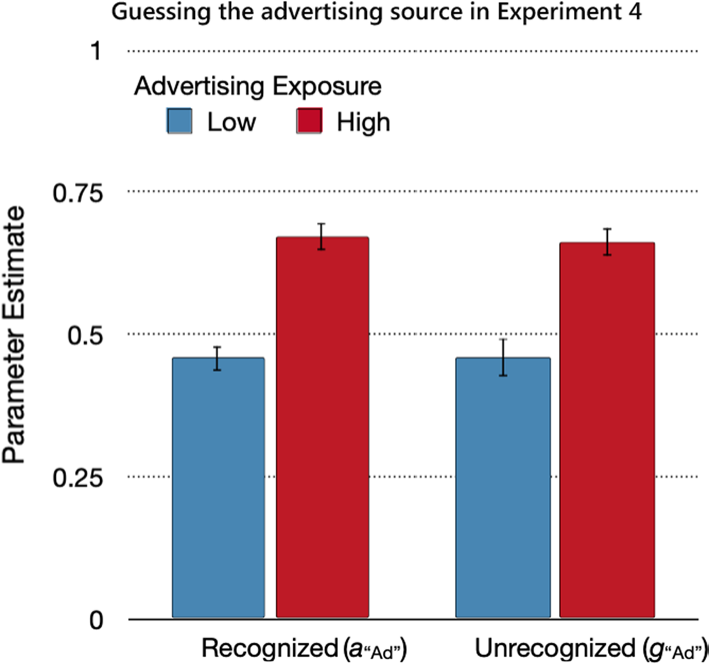
Guessing the advertising source in Experiment 4. *Note*: Parameter estimates of the parameters reflecting the probabilities of guessing the advertising source for recognized (*a*_“Ad”_) and unrecognized (*g*_“Ad”_) statements as a function of advertising exposure (low, high). The error bars represent standard errors

Guessing that a statement for which the source was not remembered belonged to the advertising or trustworthy-control sources did not differ between recognized and unrecognized statements, Δ*G*^2^(2) = 0.06, *p* = 0.97, *w* < 0.01. This equality assumption could be incorporated into a new base model for the analysis of the guessing parameters, *G*^2^(2) = 0.06, *p* = 0.97. The high-advertising-exposure condition was associated with a higher probability of guessing that a statement was from the advertising source than the low-advertising-exposure condition, Δ*G*^2^(1) = 96.97, *p* < 0.01, *w* = 0.08 (Fig. 11). The guessing bias exceeded the expected value of 0.25 when advertising exposure was low, Δ*G*^2^(1) = 256.48, *p* < 0.01, *w* = 0.13, and fell below the expected value of 0.75, albeit to a lesser degree, when advertising exposure was high, Δ*G*^2^(1) = 47.07, *p* < 0.01, *w* = 0.06.

#### Statement recognition

The statement recognition parameters could not be set to be equal for advertising and trustworthy statements because statement recognition was numerically higher for advertising statements than for trustworthy statements when advertising exposure was low, Δ*G*^2^(1) = 3.64, *p* = 0.06, *w* = 0.02, but statement recognition was numerically higher for trustworthy statements than for advertising statements when advertising exposure was high, Δ*G*^2^(1) = 3.67, *p* = 0.06, *w* = 0.02, which resulted in a significant decrease in model fit when both restrictions were applied at once, Δ*G*^2^(2) = 7.31, *p* = 0.03, *w* = 0.02. The full set of estimates of the parameters that could be derived from the source-monitoring model is reported in Table 4.

**Table 4:** Parameter estimates for item memory for the statements, source memory, and guessing as a function of advertising exposure (low, high) in Experiment 4

	Low advertising exposure		High advertising exposure	
*D*_Ad_	.46	(0.02)	.47	(0.01)
*D*_Trust_ = *D*_New_	.42	(0.01)	.51	(0.02)
*d*_Ad_ = *d*_Trust_	.31	(0.03)	.41	(0.03)
*b*	.11	(0.01)	.20	(0.01)
*a*_“Ad”_	.46	(0.02)	.67	(0.02)
*g*_“Ad”_	.46	(0.03)	.66	(0.03)

Parameter *D*_∙_ represents the probability of recognizing statements as old or new. Parameter *d*_∙_ represents the conditional probability of remembering the sources of recognized statements. Guessing parameter *b* represents the probability of guessing that unrecognized statements are old. Parameters *a*_“Ad”_ and *g*_“Ad”_ represent the conditional probabilities of guessing that recognized or unrecognized statements were labeled as advertising. Values in parentheses represent standard errors

### Discussion

In Experiment 4, participants did not judge the credibility of the product statements. Instead, participants were only required to read the statements. With respect to source monitoring, the results of the previous experiments were largely replicated. Most importantly, even when participants did not provide credibility judgments, participants’ source memory for whether a statement originated from a trustworthy or an untrustworthy source was comparatively good. Moreover, as in the previous experiments, source memory for the advertising and trustworthy sources was better when advertising exposure was high than when it was low. Furthermore, participants were more likely to guess that a product statement whose source was not remembered was an advertisement when advertising exposure was high than when it was low. As in the previous experiments, adjustments of guessing due to advertising exposure probability was asymmetric in the sense that the tendency toward guessing the advertising source in the high-advertising-exposure condition was stronger than the tendency toward guessing the trustworthy source in the low-advertising-exposure condition. These findings replicate the findings that were obtained in Experiments 1 to 3. However, there were also some differences in the results with respect to statement recognition. For example, the estimates of the statement recognition parameters are much lower in Experiment 4 (Table 4) than in Experiments 1 to 3 (Tables 1, 2, 3). We can only speculate about possible reasons for these differences. Possibly, these reasons are related to the fact that simply reading statements (Experiment 4) implies an allocation of attention to the stimulus materials that differs from what occurs when the statements have to be rated for credibility (Experiments 1 to 3).

## General discussion

High advertising exposure can pose a challenge for information processing due to the fact that advertising is often seen as an untrustworthy source of information. The perception of an advertiser’s persuasive intent may elicit coping mechanisms that aim at reducing the persuasive effect of the advertising messages (Evans & Park, 2015; Friestad & Wright, 1994). In the present study, we examined whether advertising exposure affects source monitoring. From the eyewitness testimony literature (Echterhoff et al., 2005; Lindsay & Johnson, 1989; Pena et al., 2017), we derived the hypothesis that increased source monitoring could serve as a coping mechanism for dealing with information from untrustworthy sources. However, source monitoring puts a burden on memory and it was thus not clear that people use this strategy to avoid being manipulated by advertising. The present findings provide evidence in support of the hypothesis that high exposure to advertising leads to increased source monitoring. In Experiments 1 and 2, high advertising exposure was associated with increased memory for advertising and trustworthy sources. This finding suggests that, across different contexts, increased source monitoring may serve as a coping mechanism for dealing with information from untrustworthy sources.

Given that source memory often fails (Johnson et al., 1993; Mitchell & Johnson, 2009), increased source monitoring can be only of limited use as a coping mechanism for avoiding the influence of untrustworthy sources. Simply put, it is unrealistic to expect perfect memory for the source of every single message when being exposed to hundreds of advertisements every day. It is therefore important to note that source attributions do not always reflect veridical memory for the association between a statement and its source but are often based on guessing instead (Batchelder & Riefer, 1990; Bayen et al., 1996; Bröder & Meiser, 2007). Guessing is not random because the source of information can be inferred from heuristic cues even when source memory is absent. According to the probability matching account (Bayen & Kuhlmann, 2011; Kuhlmann et al., 2012; Spaniol & Bayen, 2002), people rely on the statistical structure of the environment when making source attributions for statements whose sources are not remembered. Broadly consistent with this account, participants’ guessing behaviors in Experiments 1, 2 and 4 were strongly influenced by the high or low exposure to advertising. High advertising exposure was associated with an increased probability of guessing that the product statements were advertisements. Guessing was thus, to some extent, calibrated to the probability with which statements originated from the advertising source.

In previous studies, it has been observed that recognized statements were associated with increased credibility (Bacon, 1979; Roggeveen & Johar, 2002). The participants’ tendency to consider recognized information to be more credible than unrecognized information was particularly pronounced when recognition was only based on familiarity and source memory was absent (Begg et al., 1992; Law, 1998; Mitchell et al., 2005, 2006; Skurnik et al., 2005). Based on these findings, one could have hypothesized that, in the absence of veridical source memory, recognized statements should have a higher probability to be attributed to the trustworthy source than unrecognized statements. However, our experiments do not support this hypothesis. Instead, a more nuanced pattern of results emerged when source guessing was compared between recognized and unrecognized statements. In Experiments 3 and 4, recognized and unrecognized statements were guessed to be advertisements with equal probability. In Experiments 1 and 2, recognized statements were less likely to be guessed to be advertisements than unrecognized statements when advertising exposure was low but recognized statements were even more likely to be guessed to be advertisements than unrecognized statements when advertising exposure was high. This pattern of findings is in line with the view that the heuristic use of recognition depends on its learned validity (Unkelbach & Stahl, 2009). When having been exposed to a large amount of advertising messages, it is more likely that a product statement that is recognized but for which the source is not remembered came from an advertising source than that it came from a trustworthy source. This statistical relationship between familiarity-based recognition and source was reflected in the participants’ judgments in Experiments 1 and 2.

So far, the findings on source guessing are well in line with the well-established general principle of probability matching (Bayen & Kuhlmann, 2011; Kuhlmann et al., 2012; Spaniol & Bayen, 2002) according to which people’s source judgments reflect the statistical properties of the encoding episode. However, we also observed interesting deviations from this pattern. Specifically, Experiment 3 showed that the mere presence of advertising elicited a skeptical bias toward guessing that product statements whose source was not remembered were advertisements. In other words, the tendency toward guessing that statements whose sources were not remembered were advertising was stronger than predicted by probability matching. The adoption of a skeptical guessing strategy after exposure to advertising can be considered useful for defending oneself against advertising because this guessing strategy helps to correctly ascribe advertising messages to the advertising source. However, this skeptical guessing strategy comes at a cost: Statements from the trustworthy source have an increased probability of being misattributed to advertising. While skepticism in the face of information from untrustworthy sources may help to protect oneself from misinformation, the downside is that useful information from trustworthy sources is unjustly dismissed.

In Experiments 1 and 2, recognition of the statements from the advertising and trustworthy sources was impaired when advertising exposure was high. Given that the focus of the present study was on source monitoring, we did not postulate hypotheses about statement recognition. Therefore, we can only offer speculations on possible interpretations of this finding that can be further explored in future studies. Given that this finding was only present when participants evaluated the credibility of the product statements (Experiments 1 to 3) and absent when participants only read the statements (Experiment 4), it seems possible that the recognition decrement may be the consequence of a strategic attentional disengagement from the product statements under conditions of high advertising exposure. According to the persuasion knowledge model (Evans & Park, 2015; Friestad & Wright, 1994), an important strategy for dealing with unwanted persuasion attempts is to disengage attention from the messages which implies that the perception of a persuasion attempt “may disrupt the comprehension and elaboration of topic-related statements”(Friestad & Wright, 1994, p. 13). Such strategic efforts to cope with sources of low credibility may be more likely when participants are asked to scrutinize the credibility of newly learned information while attention may be less strategically applied and more influenced by bottom-up factors (e.g., the difference between rare and frequent stimuli) when participants do not specifically focus on credibility. Whatever the reasons, the recognition deficit under conditions of high advertising exposure underlines the conclusion that high advertising exposure can be costly for information processing.

Our conclusions are necessarily limited by the specific paradigm that was adopted in the present study. For example, in Experiments 1 and 4 we presented information from only two sources. In everyday life, the situation is often more complex because there can be many different sources with varying degrees of trustworthiness. Furthermore, we manipulated the ratio of advertising statements to trustworthy statements in a specific encoding context rather than the absolute number of advertising messages accumulating throughout the day. Also, we chose extreme ratios of trustworthy versus advertising information to facilitate the detection of differences between the low-advertising-exposure condition and the high-advertising-exposure condition. Such extreme ratios are not necessarily representative of media environments outside of the laboratory. In addition, the contents of the statements were more homogeneous than most real-world media contents. Furthermore, participants saw a large number of short statements in a relatively short time. This may be typical of situations in which consumers quickly skim through headlines and advertisements but untypical of situations in which they engage more deeply with media content. The retention interval between the encoding of the product statements and the source memory test was rather short. It can be expected that source memory is worse and guessing processes have a larger effect on source attributions when the retention interval is longer (Frost et al., 2002). Finally, source-monitoring strategies may be affected by situational and individual factors such as the participants’ mood when encountering the advertisements or the intention to learn the material. The present study should thus be seen as a first step toward identifying the source-monitoring mechanisms that are used when dealing with high advertising exposure but the robustness of the present conclusions has to be further validated in future studies.

## Conclusions

To summarize, the results reported here suggest that high advertising exposure induces increased source monitoring and an increased tendency toward guessing that statements whose source is not remembered were from the advertising source. These changes in source-monitoring processes can be seen as coping mechanisms that serve to defend oneself against excessive exposure to advertising.

## Data Availability

Supplementary analyses and data are available at https://osf.io/mdwgx/.
